# The mitochondrial genome of the deep-sea tubeworm *Paraescarpia echinospica* (Siboglinidae, Annelida) and its phylogenetic implications

**DOI:** 10.1080/23802359.2018.1424576

**Published:** 2018-01-18

**Authors:** Yanan Sun, Qianyong Liang, Jin Sun, Yi Yang, Jun Tao, Jinqiang Liang, Dong Feng, Jian-Wen Qiu, Pei-Yuan Qian

**Affiliations:** a Division of Life Science, The Hong Kong University of Science and Technology, Hong Kong, China;; b MLR Key Laboratory of Marine Mineral Resources, Guangzhou Marine Geological Survey, China Geological Survey, Guangzhou, China;; c CAS Key Laboratory of Ocean and Marginal Sea Geology, South China Sea Institute of Oceanology, Chinese Academy of Sciences, Guangzhou, China;; d Department of Biology, Hong Kong Baptist University, Hong Kong, China

**Keywords:** Tubeworm, deep-sea, mitogenome, *Paraescarpia echinospica*, next generation sequencing

## Abstract

*Paraescarpia echinospica* is a conspicuous annelid living in the cold seeps and hydrothermal vents of the Western Pacific region and relying on their endosymbiont bacteria as a source of energy and organic carbon. We report the complete mitochondrial genome of *P. echinospica*, which is 15,280 bp in length, containing 13 protein-coding genes, two ribosomal RNA genes, 22 tRNA genes and a putative control region. The overall base composition is AT-biased. The control region contains repeated nucleotide motifs. Phylogenetic analyses of the concatenated mitochondrial genes strongly support a sister relationship of *P*. *echinospica* with a clade containing *Escarpia* and *Seepiophila*.

*Paraescarpia echinospica* Southward et al., 2002 (Read and Fauchald 2017) belongs to the deep-sea tubeworm family Siboglinidae. It lacks a digestive tract but relies on the symbiotic chemoautotrophic bacteria harboured in its internal organ called ‘trophosome’ for nutrition (Rouse 2001; Bright and Lallier 2010). The species commonly occurs in the methane seeps of Papua New Guinea and the Nankai Trough, as well as the hydrothermal vents of the Okinawa Trough (Watanabe et al. 2010). Little is known about its phylogenetic relationship with other siboglinids and population connectivity. Here we sequenced the mitogenome of *P. echinospica* and explored its phylogenetic position in Siboglinidae.

A specimen of *P. echinospica* was collected from the Haima cold seep located at the northwestern slope of the South China Sea, using the remotely operated underwater vehicle (ROV) Haima in March 2016 (Liang et al. 2017), and preserved at −80 °C in the laboratory under the registration number HKUST-QIANT01. Total genomic DNA was extracted using a DNeasy Blood & Tissue Kit (Qiagen, Halden, Germany) and used for whole-genome sequencing on an Illumina Hiseq (2 × 150 bp Pair-end reads). Approximately 30 Gb sequence data were assembled *de novo* using SPAdes v3.9.1 (Bankevich et al. 2012) and the contig of mitogenome was verified by BLASTN (Altschul et al. 1997) using the mitogenome sequence of *Escarpia spicata* (Li et al. 2015) as the query sequence. Gene annotation was performed with MITOS web server (Bernt et al. 2013). The sequence has been deposited in GenBank under accession number MG462707. A maximum likelihood (ML) tree was constructed using the IQ-TREE web service (Trifinopoulos et al. 2016).

The circular mitogenome of *P*. *echinospica* is 15,280 bp in size, with an overall base composition of 30.11% for A, 22.77% for C, 12.91% for G and 34.21% for T. The genome exhibits codon biases, with an AT content of 63.15% in protein-coding genes. The mitochondrial genome contains 13 protein-coding genes, two ribosomal RNA genes and 22 tRNA genes. The gene order is identical to that of reported siboglinids (Jennings and Halanych 2005; Li et al. 2015). ATG is the start codon for all genes. Most genes use either TAA or TAG as the stop codon except four genes (*nad2*, *cox1*, *nad6* and *cob*) which use a single T as the stop codon. A 593 bp control region lies between *trnR* and *trnH*. The control region contains two types of simple repetitive motifs: (TA)_n_ has been found in all reported mitogenomes of siboglinids (Li et al. 2015), whereas (ATATATGTGT)_n_ is unique to *P*. *echinospica*.

The phylogenetic analysis based on *P*. *echinospica* and mitogenetic sequences of all 14 species of siboglinids uploaded on GenBank indicates that *Paraescarpia echinospica* is sister to a clade comprised of the cold seep siboglinids *Seepiophila jonesi* and *E. spicata* (Figure 1). Based on these data, primers for individual genes can be designed to study the population connectivity, which will help the conservation of these deep-sea animals that are facing increasing human activities such as trawling and mineral extraction (Mengerink et al. 2014).

**Figure 1.**
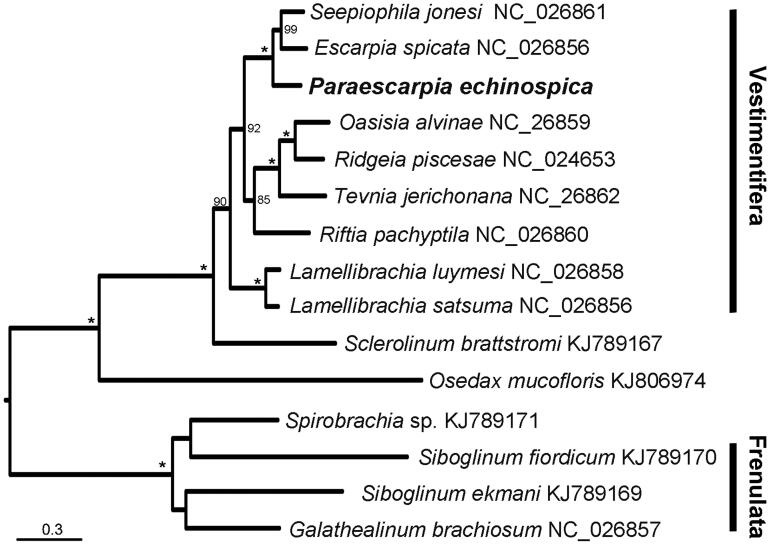
The maximum likelihood (ML) tree of 15 species of Siboglinidae based on the concatenated nucleotide sequences of 13 mitochondrial protein-coding and two ribosomal RNA genes. The number at each node is the bootstrap support value. Asterisks indicate bootstrap support value =100. The number after species name is the GenBank accession number.
